# Weaving a vicuña shawl

**DOI:** 10.1186/s13570-022-00260-6

**Published:** 2022-11-24

**Authors:** Bibiana Vilá, Yanina Arzamendia

**Affiliations:** 1 VICAM: Vicuñas, Camélidos y Ambiente, Luján, Argentina; 2 CONICET: Consejo Nacional de Investigaciones Científicas y Técnicas, Buenos Aires, Argentina; 3 Universidad Nacional de Luján, Departamento Ciencias Sociales, Luján, Argentina; 4 INECOA: Instituto de Ecorregiones Andinas, CONICET- UNJu: Universidad Nacional de Jujuy, San Salvador de Jujuy, Argentina; 5 Facultad de Ciencias Agrarias, Universidad Nacional de Jujuy (UNJu), San Salvador de Jujuy, Argentina

**Keywords:** Artisanal, Fibre, Indigenous, Puna, Argentina

## Abstract

Vicuñas (*Vicugna vicugna*) were at risk of extinction due to indiscriminate hunting for their fibre in the mid-twentieth century. The conservation of the species included numerous international and regional legal mechanisms, as well as the will and care of local communities. The vicuña was saved, and now it is classified as “Least concern” by the IUCN Red List of Threatened Species. Sustainable harvest of vicuña fibre is achieved by way of the *chaku*, capture and live-shearing (an ancient practice, now informed by modern knowledge of biology). Although most communities and cooperatives sell raw fibre, prices are falling. The making of artisanal vicuña garments has been identified as an activity that potentially leads to sustainable development in the Andean Altiplano. This paper analyses a key aspect to achieve this goal, a detailed description of the production (including economic and time costs) involved in weaving a shawl. The manual work has been recognized, with an hourly rate and costs calculated. The first action before starting any intervention was a ceremony, in which the family women infused the fibre with the smoke of a local plant, *k’oa* (*Parastrephia* spp.). The rituals and traditions are an important part of livelihoods in the Andes. The steps to creating the finished garment were fibre acquisition, dehairing and cleaning the fibre, spinning, twisting, loom weaving, finishing and fringing. The techniques for spinning and weaving are an essential part of the indigenous cultural heritage; no intervention or suggestion was made in that regard. The final cost of the garment was approximately 3,300 US dollars and half of this cost was the dehairing and cleaning of the fibre.

## Introduction

The vicuña (*Vicugna vicugna*) is a wild South American camelid, the smallest of the family *Camelidae* (Franklin 2011). The species live in high altitude of more than 3500 masl, in arid and semi-arid steppes in the *Puna* or *Altiplano* of Argentina, Chile, Peru and Bolivia. The species was also introduced in Ecuador. Vicuñas have one of the finest and most expensive fibres in the world with a mean diameter of 12 µm and an approximate price of 400 US dollars/kilo.

The relationship between wild vicuñas and Andean people can be traced back to the peopling of America, where the first settlers were vicuña hunters (Yacobaccio 2009). This “Coupled Human and Natural System” or CHAN (Liu et al. 2007) lasted approximately 10,000 years, leaving profound legacy effects—impacts that human-nature couplings have on later conditions (Liu et al. 2007). The good and bad moments for vicuña population dynamics are closely linked to human activities.

### Phases of “Coupled Human and Natural System: The vicuña and the altiplano”

The phases of this CHAN can be summarized: (a) vicuñas as prey for hunters in the prehistoric period (10,500BC–AD 1470); (b) the prehispanic sustainable use during Inca times (1470–1535) in the Tahuantinsuyu by *chakus* consisting of a capture with a proportion of animals killed and most of them sheared and released, where vicuñas populations were able to maintain their numbers; (c) the colonial period with the slaughter of vicuñas by their thousands that continued during the Independence period (1810–1950) that sent the species to the point of extinction by the middle of the twentieth century (Yacobaccio 2009); (d) the strict conservation phase (1960–2000) and the recovery of the populations, explained in detail below; (e) the 2000 neo-*chakus*: several populations in all the countries with vicuñas managed for a non-lethal use consisting in the harvesting of the fibre by capture and shearing live animals; (f) current times in which research is needed to address the sustainability of the large-scale use of the species, in particular the challenges for animal welfare and poaching.

### Vicuñas conservation and management

The recovery of vicuñas was a successful story with many actors: from a global population estimated at 14,500 animals in 1960 (Laker et al. 2006), a strict conservation period started which had a time lag—time between the human-nature interactions and the appearance of ecological and socioeconomic consequences (Liu et al. 2007) of two to three decades for the recovery of several populations of the species. The main legal instruments which secured the resurgence of vicuñas were the Convention for the Conservation of the Vicuna, signed in La Paz in 1969, then replaced 10 years later by “Convention for the Conservation and Management of the Vicuna” ratified by Argentina, Chile, Peru, Bolivia and Ecuador. This Convention in its Article 1 stated: “The Signatory Governments agree that conservation of the vicuna provides an economic production alternative for the benefit of the Andean population and commit themselves to its gradual use under strict State control, applying such technical methods for the management of wildlife as the competent official authorities may determine” (ECOLEX 2022). Also, it was very important the Classification of the species in CITES appendix 1 “Appendix I includes species threatened with extinction. Trade in specimens of these species is permitted only in exceptional circumstances” (CITES n.d.), and as Vulnerable in 1982 in the IUCN Red List of Endangered Species (IUCN 2022). Those measures were accompanied by regional, national and provincial policies regarding the species including the creation of reserves and protected areas, specific conservation legislation and prosecution and punishment of hunters. It should be especially noted that the conservation of this species relied on the commitment and work of the IPLCs (indigenous peoples and local communities) who were and still are key actors in the territory.

The recovery was sustained, several populations increased in numbers and the vicuña is now classified as “Least concern” in the Red List and “Moderately depleted” in the Green Status Assessment (Acebes et al. 2018) of IUCN. During the last decades, most populations changed to CITES ap II “Appendix II includes species not necessarily threatened with extinction, but in which trade must be controlled in order to avoid utilisation incompatible with their survival”. This new situation led to several projects in the four countries that included the use of vicuñas “by live shorn animals” the only way CITES allows, including a diversity of management regimes (Lichtenstein and Vilá 2003; Acebes et al. 2018). One of the main debates in the recovery of the species was the alternative of captive vs wild management of the species. Lichtenstein (2006) analysed the captive management of vicuñas in Argentina in detail and concluded that these systems failed in several aspects: they did not contribute to reducing poverty; the profitability was low; it indebted the beneficiaries; there was little reproduction of the vicuñas in confinement. She concluded that captive management was not a good tool for the conservation of the species. Currently, there is still captive management in INTA Abrapampa, Argentina, and in modules of 700–1000 ha in Peru. In the other countries, the main management of the species is in the wild.

### Wild vicuñas management in Argentina

The wild management of vicuñas in Argentina started in 2003 in Cieneguillas, Jujuy, with the first capture by the MACS (2022) (Sustainable economic utilisation of wild South American Camelids: Strategies for improving rural productivity in pastoral communities in Latin America) team project. This initial work in the country was generated from the demand of a group of associated producers in Cieneguillas and developed during three consecutive years with vicuña captures, from which the producers obtained 67 kg of fibre. The researchers of the MACS project studied the effects of capture and shearing on animal welfare and other biological parameters, reported in two books, one in Spanish (Vilá 2006) and the other in English (Gordon 2009), and several papers (Vilá 2002; Yacobaccio and Vilá 2002; Lichteinstein and Vilá 2003; Vilá et al. 2004; Arzamendia et al. 2006; Arzamendia et al 2010 and 2012; Vilá et al. 2010; Marcoppido et al. 2010; Arzamendia and Vilá 2012, 2015). After the end of the project, several MACS members founded VICAM *(Vicuñas, camélidos y ambiente*) and sustained most of the MACS’ lines of research.

In 2012, a new series of captures began in Santa Catalina in joint work with the local cooperative COOPASAC (Santa Catalina Agro-livestock Cooperative). Based on the demand of many other communities, a manual specially written for IPLC was published (Baldo et al. 2013) in order to share and extrapolate experiences and procedures for the expansion of vicuña management carried in other places with different institutional technical advice. What seemed like the most challenging objective, which was to capture vicuñas and obtain their fibre in *chakus* with high animal welfare standards, was a real possibility presented in that manual that was (and still is) accessible free of charge from the Internet. These scientific results of the sustainable use of vicuñas, added to those of the MACs, were the basis for legislating on the management of vicuñas in the province of Jujuy. The researchers responsible provided advice to the Jujuy authorities and are the authors of the guidelines (Arzamendia et al. 2012), which were a key input for the province’s vicuña law. In 2014, the government of Jujuy initiated a policy of vicuña management and promoted the creation of an association of communities, the “CAMVI” (Andean communities that handle vicuña) involving several technical advisory institutions.

### Sustainability of vicuña use

A recovered species, with sustainable management with shearing, carried out by indigenous communities, could have an invaluable green market niche. But the enormous difficulties faced by the communities in relation to bureaucracy, the value chain and marketing with tremendous inequity in a global market (Sahley et al 2004; Lichtenstein 2010) hinder the possibilities of local development through the sustainable use of vicuñas. In an oligopsony/monopsony (few or one buyer), the commercialization of raw fibre is not solving poverty in the Andes (Stollen et al. 2009; Lichtenstein 2010). Fibre prices are variable (and currently decreasing below 400 dollars per kilogramme), and the relationship between the percentages earned by Andean communities in the retail price of a garment is only 2–6% (Kasterine and Lichtenstein 2018).

With this scenario, the alternative of producing high-quality handmade garments is a potential solution, if sufficiently regulated. In Argentina, Catamarca province has a tradition of weaving vicuña, especially ponchos (Rolandi 2006), including a government fund for the sale of subsidized fibre to artisans (Castilla et al. 2021). In Jujuy, the activity is incipient with the CAMVI group, offering vicuña garments in their web page (Camvi 2022).

We consider that it is absolutely fundamental for thinking and designing policies that include the sale of handicrafts, to carry out a detailed study of times and costs and people’s perceptions in relation to this activity. That is why our team, which until now had studied the ecology, captured vicuñas and worked towards sustainable scenarios, went one step further and decided to study in detail the process of weaving a vicuña shawl. The objective of this work is then to make a description of the steps, the costs and perceptions in making a beautiful and fine vicuña garment.

### Study area

The town of Barrancas is located 23° 20′ 30.75″ South, 66° 05′ 25.37″ West, a high altitude (3600 masl) semi-desert with a mean summer precipitation of 180 mm/year, in the right margin of Barrancas River, a tributary of Las Burras River, which drains into Salinas Grandes basin. The area is renowned for its several archaeological sites which are protected in a declared natural and cultural reserve by the town authorities. The town of about 350 inhabitants has a primary and secondary school, first aid room, an archeological museum, library and sports centre.

The locality, including the rural areas, comprises about 1300 people, in two indigenous communities and a neighbourhood centre. The main activity of the areas is pastoralism of sheep and llamas and subsistence agriculture. State work and state subsidies are also an important source of income. In the town, there are numerous artisans who work with llama fibre.

## Methods

We carried out this work from April 2021 to April 2022 together with a family in the Puna, in the town of Barrancas (also known as Abdon Castro Tolay). In particular, this family was chosen and invited to co-create the project because one of its members makes superb weaving of “barrancan” cloth in llama fibre. We obtained funding from the Institute for Global Environmental Strategies, regarding the implementation of the Satoyama Development Mechanism (SDM) from Japan for the project “Recovery and use of camelids and their fibre as potential resources to improve local livelihoods in a post-pandemic scenario in the Andean Altiplano” that included the possibility to get stipends to pay hours dedicated to research-action as none of the people involved had previous experience with vicuña fibre. The Barconte family has inhabited the area since the foundation of the town and its members divide their activities between llamas and sheep herding, agriculture, llama fibre handicrafts and guide services in the archaeological museum and archaeological sites. They have experience with llama fibre but they did not work with vicuña fibre before. We have their individual permission to use pictures and recordings. We worked under the code of Ethics for research, research-action and ethno-scientific collaboration in Latin America (Version two) of SOLAE, the Latin-American Society of Ethnobiology.

### Procedures

The steps of the project included sourcing, processing, product development and analysis. In each step, we recorded with mixed methodology, as we handle quantitative and qualitative data and their interactions (Albuquerque and Lucena 2004).

1. Purchase of fibre: We bought from the Santa Catalina Agro-livestock Cooperative (COOPASAC, register as INAES 49,350), 3.052 kg obtained from the vicuñas in the Carayoc capture on the 18/11/2018 approved by Res 088/2018 as part of the Local Management Plan (Arzamendia et al. 2014) by the Provincial Directorate of Biodiversity of the Ministry of Environment of Jujuy. Individual bags number 1, 2, 4, 5, 6, 7, 9, 10, 11, 12, 13, 14, 15, 16, 18 each corresponding to a marked vicuña. Part of this fibre was used in this project.
2. Weight of the dirty raw fibre in each bag.
3. Record hours of cleaning (removing all plant debris and litter stuck to the fibre) and dehairing (removing of thick fibres) of each bag.
4. Weigh the dehaired clean fibre. Calculate the yield of the raw material.
5. Yarn: record of the number of hours it takes to spin the amount of yarn needed for a shawl.
6. Twisted yarn: record the number of hours needed for twisting the yarn.
7. Loom weaving: record the number of hours needed to weave the shawl.
8. Conditioning of the garment. Cleaning and conditioning the fringes.
9. Record of feelings of the fibre cleaner, spinner and weaver. Comparison between the sensation of weaving vicuña and llama.

In individual notebooks, each of the members of this project registered the date and the number of hours spent on one of these tasks: cleaning, spinning, twisting, or weaving. Together, we carried out a participatory survey (focused on group discussion of each activity), to check their notebooks and identify the main criteria used by the artisan in each step of this project.

To calculate the costs associated with this work and to reward the work honestly, participants were asked to propose what they considered a fair price per working hour. The agreed value was 350 Argentine pesos per hour, the equivalent of approximately 3.5 USD  for cleaning, spinning and twisting the fibre at the time of the arrangements. This quantity exceeds the amounts paid in the area, for example a hotel receptionist earns (Argentinean pesos) 200 (2 USD) per hour. Loom weaving was paid 520 pesos (5.20 USD) per hour. The funds were managed as stipends given the condition of a unique case study.

## Results

### Sourcing

The vicuña fibre was acquired during a visit to the area in April 2021 at the Santa Catalina Agro-livestock Cooperative. We bought 3.052 kg, paid 450 USD per kilogramme, a total of 1373.4 USD or 130,473 Argentine pesos in the official rate at that time (95 Arg pesos $/1USD 21th April 2021).

We took six bags of the fibre and carried it to Barrancas. Together with the Barconte family, we weighed, with a precision balance, the fibre and talked about the task to be carried out. The assignment of tasks within the family was as follows: America and Lis that are sisters cleaned the fibre, Norma (their mother) spun and twisted and Eusebio, who is Norma’s brother, wove on the loom. We agreed with the family the hourly rate of 350 Arg pesos (approximately USD 3.50 per hour) for all tasks except the loom and 520 Arg pesos (approximately USD 5.2 per hour) for each hour of work on the loom. No artisan who works with llama fibre in the area charges by the hour, since they are self-employed.

### Processing

**Fig. 1:**
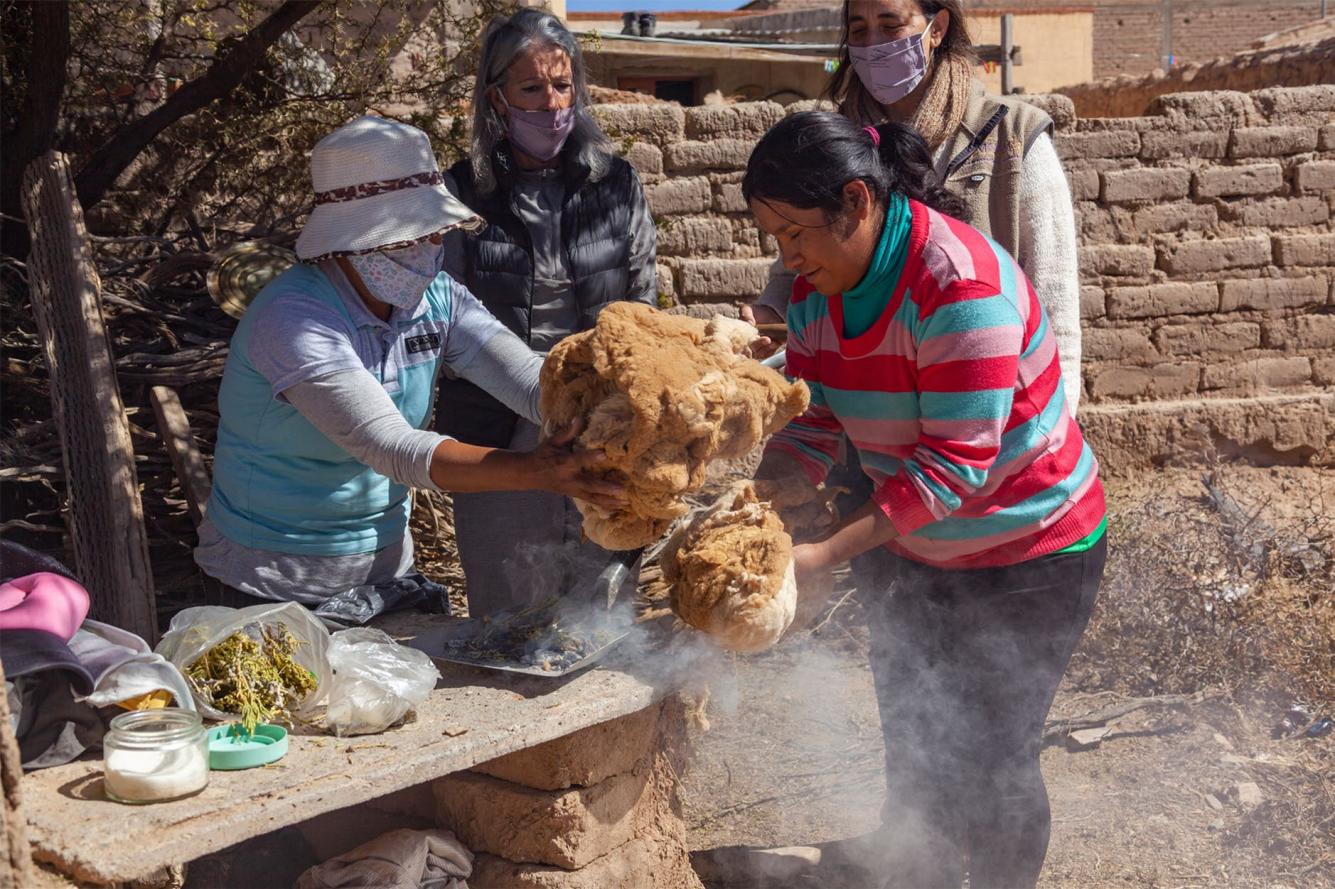
Ceremony of the fibre smoking. From left to right: Norma Barconte, Bibiana Vilá, América Barconte and Yanina Arzamendia. Photo by Silvina Enrietti

As soon as they received the fibre, America and Norma told us that before doing anything with it they needed to make a ceremony to “smoke to take the air off” the fibre. America and her mother performed the smoking ceremony using “k’oa” (*Parastrephia* sp.), a resinous aromatic high-altitude plant (Fig. 1). “*The smoking is very important for us because vicuñas’ wool in the Andean world has “air” [es airienta] because it is in contact with nature as a wild animal and if we take into account that negative energy and positive energy exists, surely the animal, like us, goes through those places, and smoking then is like a protection towards the person who is going to do the handling of the wool, the cleaning, the dehairing, the spinning and the weaving” (America 8–12). “You have to smoke llama wool and vicuña wool more…it's like that…and people used to say like that, it's that you shake the powder has the smell of the vicuña, that little smell of vicuña*” (Eusebio 174–8).

In the first meeting of the whole group, Eusebio expressed his interest in weaving by combining the two colours of vicuña fibre (light and dark) in a “partridge eye” pattern. America and Norma also commented: “*We are going to have to be careful to note the time because we are never looking at the clock, now we are going to do it*” (America 40–4).

#### Cleaning

*For me it was nice, let’s say, I* *had never done it before and it did cost us the first vicuña because we didn’t know how to start and we hadn’t taken out all the trash, the little seeds, everything it has (…) That is one of the experiences. Then at the end I was already more practised *(America 4-12).

*Cleaning the fibre is complicated because you have to sit down, you can't do it in the field, because of the wind, and because you have to watch the llamas. You have to remove all the little sticks and that is the most difficult thing along with the bristle. The heaviest work, because there is no progress. I sit here and in two hours I'll do a little thing because I'm not moving forward because I have to be with the tweezers pulling out each stick. If you shake it, it doesn't fall out, and if you shake it a lot it falls apart. Eusebio says that he, in the row, can be taken out, but not so much. This little piece takes two hours, three hours* (America 18-11).

*I want to try vicuña because I already know how to clean llama fibre, the vicuña seems to be more difficult, finer, softer and shorter* (Lis 8-9).

#### Spinning

*The truth is that I found spinning the first vicuña difficult at first. That is one of the experiences. Then, in the end, it’s like I was more practiced and it was nice*. (Norma 3-12)

*I changed my pushka* [wooden hand spindle] *because before I had a bigger pushka that was thicker and heavier and now I have a lighter, finer one for the vicuña. I spin and it comes out, you have to spin slowly because if I stretch it, it breaks and you have to do it slowly, with the shortest yarn. With this fibre, if you shake it is cut*. (Norma170-8)

*As the yarn is being spun, the “filling” of the pushka is made and when the thread accumulates, it is called “the filling*” (Norma 44-4)

*It is the first time that I am spinning vicuña. At first, it was difficult, the first vicuña, and now I am a little more expert. The longest fibre is spun better and faster and the shorter parts were trickier. The short wool is because to shear the live animal you have to leave wool. It is not the problem of the shearer, the shearer is going to shear as it is the wool, is not going to remove everything, it is not going to kill the vicuña, the belly is shorter, it is more compact, it is tighter, it has more soil, it is the part where it lies down, where it crushes the wool and it is the one that costs you the most. It tires my eyes; I wear the tears that you brought me* [Norma complained about red eyes]. *The people who get it* [the fibre] *from the skin from poaching have longer fibre but it is prohibited*. (Norma 19-11)

#### Weaving

*Let them spin all the yarn, when I start weaving on the loom I have to have all the thread done, when I start I have to be able to continue to the end. I will make a looser weave, the “partridge eye”. The lighter the warp and the darker the weft.* (Eusebio 174-8)

*The loom is a square loom with four hangers (…) I bought the loom in Abrapampa at the Puna Cooperative; from there I won a project and bought a loom and the llama fibre spinning machine. First, I wove a lot, but later it couldn't be sold and that's how paralyzed I was. First you have to make the warp, then you have to pass the reed and then put the held wires and stretch well and regulate everything so beautifully and just start weaving. What takes more time is to pass the thread through the reed, everything must be sliding*. (Eusebio 6-9 12)

#### Differences between vicuña fibre and llama fibre

*The llama spins faster because the wool stretches more and is longer, while the vicuña is softer and you have to treat it with more care, it is more delicate*. (America 4-12)

**Fig. 2:**
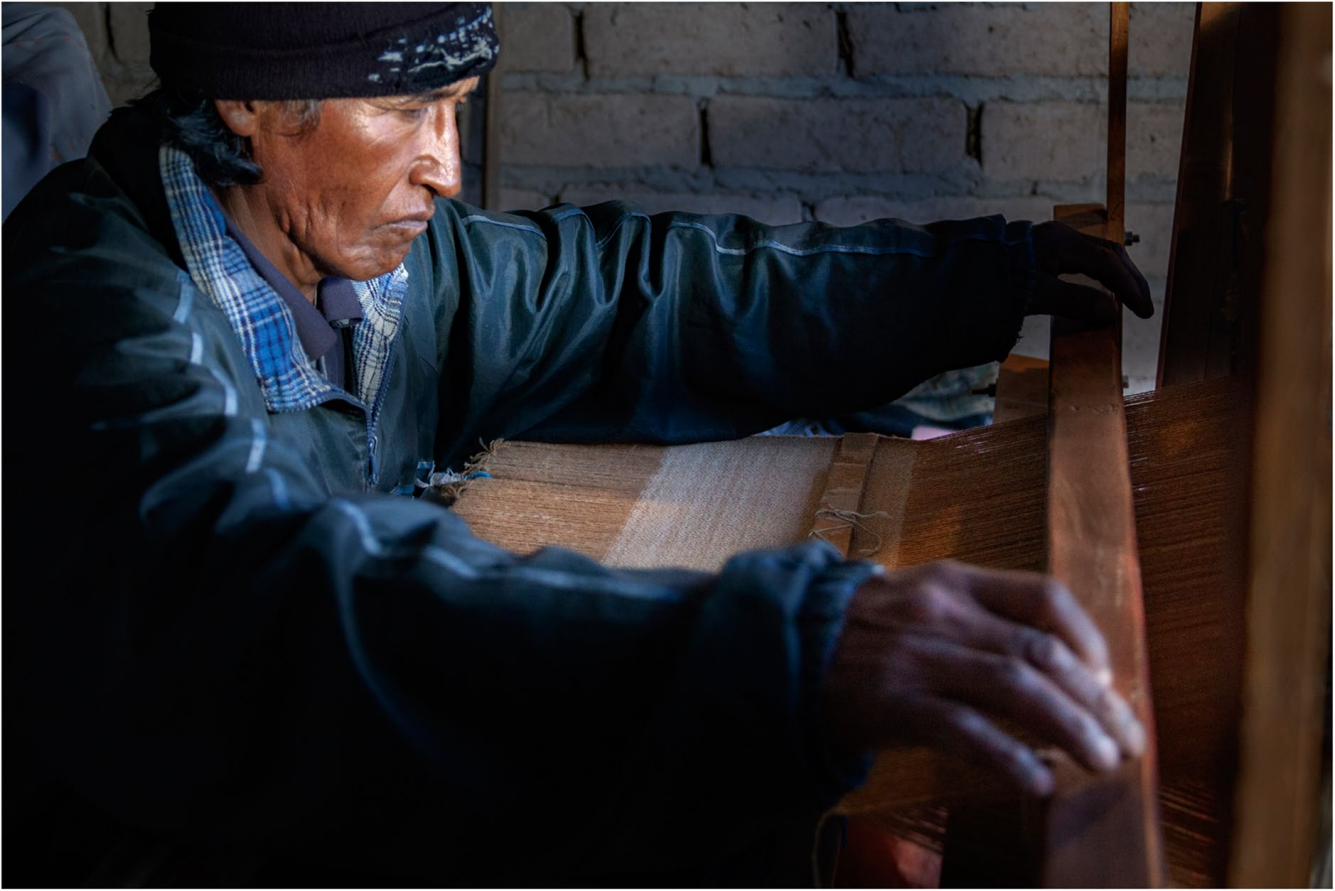
Eusebio weaving in the loom. Photo by Silvina Enrietti

In the loom: *The vicuña has almost no difference from the llama, it is much softer, but after that everything is fine, it is a good nice fibre* (Eusebio 11–12) (Fig. 2).

#### General comments

*In the Pandemic, we artisans didn’t sell anything because you couldn’t go down to the “Quebrada de Humahuaca”, and there weren’t any tourists in the quebrada, we didn’t sell because nobody came*. (America 39-4)

[it is good] “[this project…] *Paying well paid hours. The common craftspeople do not charge by the hour, nor do we write down how many hours we work. We do this badly, to have more or less, we don’t know how long it takes us* [to work with llama fiber]. (America 8-12)

[the problem is getting the yarn]. *If I had the yarn I could do it, I don’t have any work so I could knit*. (Eusebio 20-11)

*In a Facebook group of weavers, there are vicuñas garments from Catamarca...they publish good things. If I had a yarn, I would do this* [she shows a picture of a vicuña’s sweater] …. (America 20-11)

### Production of the garment

A shawl 180 cm long and 52 cm wide was woven combining the vicuñas’ dark (on the back) and light (from the side and ventral areas) fibre of the animals. The detailed process including fibre weight, working hours and performance (% of yarn of the total of raw fibre) is presented in Table 1.

**Table 1:**
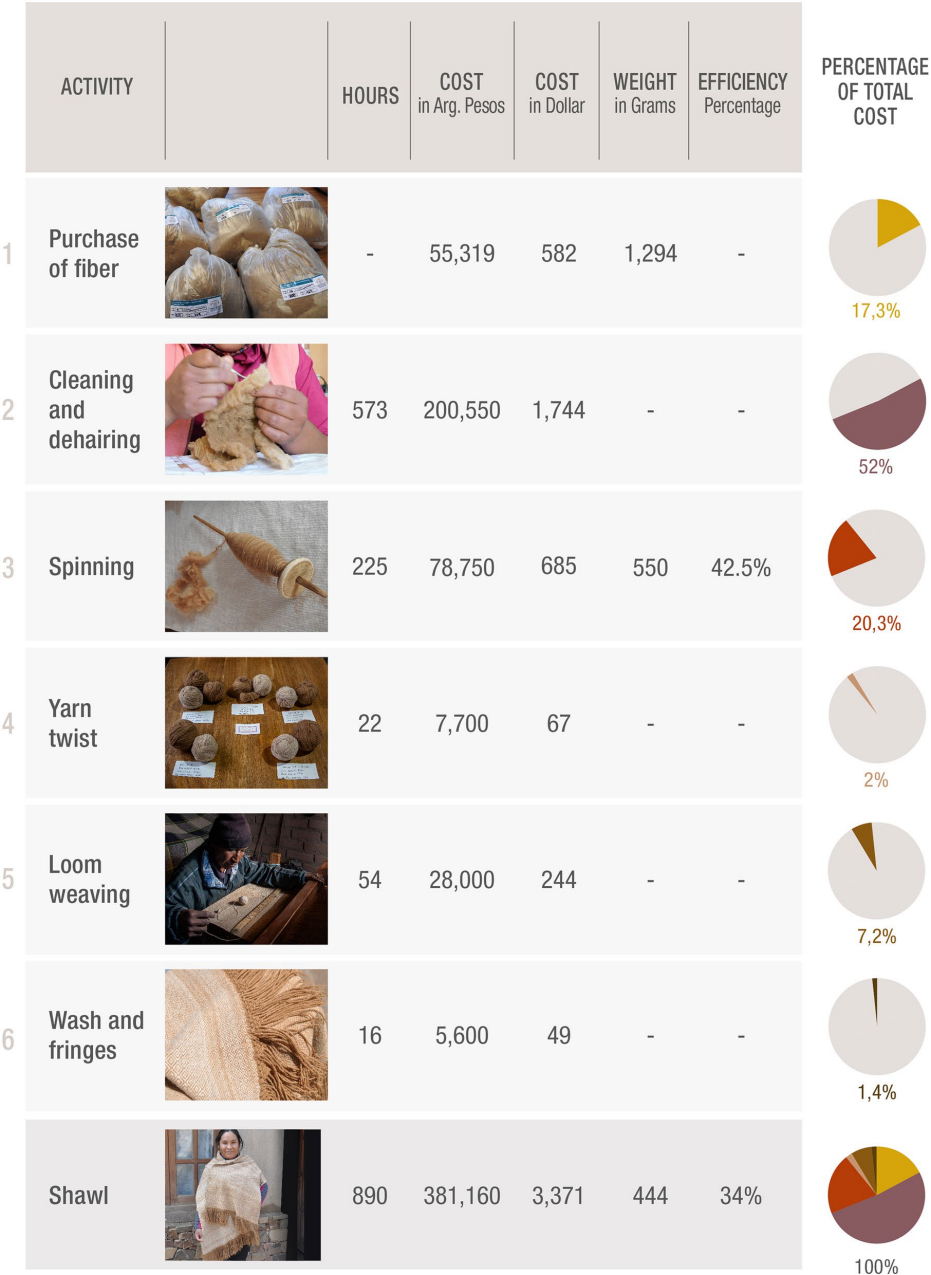
Description of the steps from vicuñas raw fibre to a shawl, including images of the activities, number of total hours, the cost in pesos and dollars (please observe that the rate in the purchase of the fibre was that one at that date 95 pesos per US dollar, while the rate for the rest of the table is the current while writing the paper 115 pesos  per US dollar). Photograph credits: Silvina Enrietti, Yanina Arzamendia and Bibiana Vilá

The analysis of the complete process showed a low efficiency as only 34% of the raw fibre ended in the shawl (Table 1). The costliest and time-consuming point was the cleaning of the fibre, which was also frustrating for the sisters who took care of that, due to the slowness of the process and therefore to the delay of an observable result after hours of work (see America 18–11).

The main cost of the shawl (more than 50%) was the preparation of the fibre for spinning. In fact, very few people were willing to do this work, and America and Lis did it because their mother was going to spin the fibre. Spinning was the second important cost (20%) followed by the raw fibre (16%). Contrary to our expectations, loom weaving was very precise and fast (Fig. 2).

## Discussion

South American camelids, as a local breed and endemic fauna, contribute with important materials, particularly fibre, to the improvement of pastoral livelihoods both for the present needs and future challenges (LPP et al. 2010; IPBES 2022).

We were able to follow the path from the wild vicuñas that we studied for several years, and their fibre obtained by live-shearing to a woven shawl in a process that included the involvement of an indigenous family of artisans.

The ceremony as the first step was imbued with sacredness and had the objective of removing the “wilderness or air” of the fibre. In the Andean cosmovision, the “air” that enters a person can be a causative factor of ailments, especially the “fright” (Strasser 2015) and there is an association between wild species and places with the “air”, that must be removed from the fibre with *k’oa* smoke to be able to handle it with ease. Infusion with the smoke from aromatic plants is one of the essential components in Andean ceremonies, perhaps the most important, and dates back to preHispanic ancient times (Mariscotti 1978; Santisteban 2020).

This weaving activity was conceived as a potential alternative to improve local livelihoods in a post-pandemic scenario, a possible economic-cultural solution to the post-COVID-19 crisis. This is why the economic recognition of the work and its neat record was so important, being the fair payment a central component of the project. Analysing the difficulties for selling the fibre by the communities, the downward trend in international prices and the potentiality to improve benefits, the “adding value to the fibre” always appeared in publications as a way to overcome this issue (Lichtenstein 2010; Cooney 2019; Vilá et al. 2020). In fact, the resolution 392 of the XXXIII meeting of the Vicuña Convention in 2017 explicitly suggests that each signatory country carries, to the extent possible, studies on the vicuña fibre value chain, including at least the following aspects: production, conservation, marketing costs, control and traceability, characterization of the fibre, the study of the market and search for alternatives.

The traceability of garments is a mandatory issue to avoid the use of fibre from poaching. Traceability of legal artisanal garments is possible by controlling the obligation to use legal fibre, a register of artisans and the knowledge of percentages of efficiency. However, Castilla et al. (2021) point out that “there is no system in place to trace added-value products such as dehaired fibres, fibre selected by colour, spun fibre and various types of garments”. The potential of vicuñas as an option for developing environment-friendly and value-added products from SEPLS that equitably benefit local smallholders is high and possible (Takahashi et al. 2022).

Although it is a single-case study, and all the participants were novices and inexperienced in working with vicuña fibre, we do not have any information from a similar study including fair prices for labour and a detailed description of the time and steps needed. It was clear from the beginning of the project that the Barconte family took it more as a nice extra and risky activity than as a possible source of secure work in the future, probably due to the lack of initial capital to buy the fibre by themselves.

### Some limitations and challenges

Two problems were detected after the experience; the first one was the low performance in relation to the quantity of raw fibre. The “short fibre” is a component of the discard and can be partially avoided with more careful shearing or using a shearing machine. The “dirty” and compacted fibre is unavoidable as vicuñas roll every day in sand wallows (Vilá 2012).

When fibre production quantities are low, such as the case of vicuña, generally dehairing is done in the manual form, and that experience give yields around 70% (Adot et al., 2008). Although some authors consider this activity as a new source of employment and economic income (Siguayro, 2009), in our work, this activity was not pleasant for the women who did it, mainly because it was tiring and with very low results over time. If we consider the percentage that the cleaning represents in the final price (more than 50%), it is important to find ways to decrease it. Dehairing machines for vicuña fibre exist (Textil los Andes SA 2022), and there is the possibility to access this service (Lamas pers. comm.), as the main processes in which people can assert their traditional knowledge and culture are spinning and weaving. In reality, the shawl price of more than 3,000 US dollars is an uncomfortable outcome of this case study. Potential wealthiest buyers tend not to choose handmade garments (they prefer the industrial high-profile brands of vicuña garments) and the chance of encountering a wealthy person (with such an amount in cash) and an indigenous fibre weaver in the same place is almost nil. Therefore, the existence of intermediaries greatly complicates the picture if the initial value of the garment is so high and local communities have poor possibilities to negotiate. In that case, the cost of labour is usually the variable that can be adjusted, leaving artisans working for low returns and low incentives.

One of the issues that must be taken into account in this case study is that all the participants in the handling of the fibre had no previous experience with vicuña fibre. With practice and experience, efficiency is likely to increase. Another problem is that it is very difficult to make economic planning, in a region with high inflation and devaluation of the national currency.

#### Policy recommendations

Some policy recommendations include the following: to modify the two stages that are costly in terms of labour efficiency - the manual shearing and dehairing/cleaning - by mechanizing the processes. It would be helpful for the people involved in the processes to earn their money at the time they are working, and not that their payment being deferred at the time of sale of the garment. It will be necessary to reach wealthy people with green and sustainable intentions through intermediaries who do not detract from the value of the crafts that are then fair for the communities. Perhaps the creation of a transparent mechanism of e-commerce managed and supervised by the communities.

## Conclusions

This weaving activity was conceived as a potential alternative to improve local livelihoods in a post-pandemic scenario, economic recognition and fair payment being a central component of the work. All the processes of making the shawl were carried out with the knowledge of the indigenous family, without the intervention of the researchers who fulfilled a role of facilitators. The shawl cost was high, more than 3,300 US dollars, and half of the production cost went to the cleaning and dehairing activity which was also the only activity that was not pleasant for the women. In general, in all other activities, including spinning and weaving, people said they felt comfortable and that the fibre was more difficult but softer, and more delicate.

## Recommendations

Given our result, it would be interesting to analyse the use of sharing and dehairing machines to avoid the costliest section in terms of time and money and improving efficiency. Considering the value and work of producing the garment, it is vital that before embarking on these activities, the people involved adopt a sales strategy to improve access to potential buyers.

## Data Availability

All data generated and analysed during this study are included in this published article [and its supplementary information files].
